# Supplementary material for: Netcombin: An algorithm for constructing optimal phylogenetic network from rooted triplets
Source: PLoS One. 2020 Sep 18;15(9):e0227842. doi: 10.1371/journal.pone.0227842 (PMC7500971; doi:10.1371/journal.pone.0227842)
Supplement: S4 File — (ZIP) [file pone.0227842.s004.zip › SequenceData-Triplet/sequenceData-10-20-30-0-Triplet/length 200, numseq 40, rate 10^-7/7 seq.pdf]

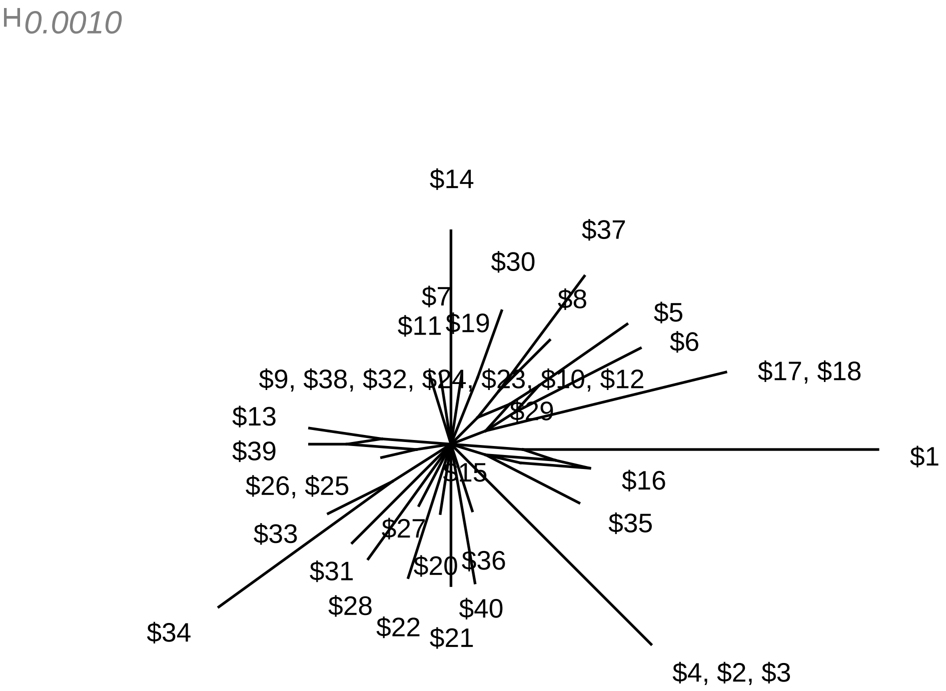
0.0010
$14
$37
$30
$7
$8
$5
$11
$19
$6
$17, $18
$9, $38, $32, $24, $23, $10, $12
$29
$13
$39
$1
$15
$16
$26, $25
$35
$33
$27
$31
$20
$36
$28
$40
$22
$21
$34
$4, $2, $3
